# Positive role of cell wall anchored proteinase PrtP in adhesion of lactococci

**DOI:** 10.1186/1471-2180-7-36

**Published:** 2007-05-02

**Authors:** Olivier Habimana, Carine Le Goff, Vincent Juillard, Marie-Noëlle Bellon-Fontaine, Girbe Buist, Saulius Kulakauskas, Romain Briandet

**Affiliations:** 1 Unité Mixte de Recherche en Bioadhésion et Hygiène des Matériaux, INRA-ENSIA, 91744 Massy cedex, France; 2 Unité Bactéries Lactiques et pathogènes Opportunistes, Institut National de la Recherche Agronomique, Domaine de Vilvert, 78352 Jouy-en-Josas cedex, France; 3 Department of Molecular Genetics, Groningen Biomolecular Sciences and Biotechnology institute, University of Groningen, Kerklaan 30, 9751 NN Haren, The Netherlands; 4 U781, INSERM, Hôpital Necker enfants malades, Tour Lavoisier, 149 rue de Sèvres, 75015 Paris cedex, France; 5 Department of Medical Microbiology, University Medical Center Groningen and University of Groningen, Hanzeplein 1, P.O. Box 30001, 9700 RB Groningen, the Netherlands

## Abstract

**Background:**

The first step in biofilm formation is bacterial attachment to solid surfaces, which is dependent on the cell surface physico-chemical properties. Cell wall anchored proteins (CWAP) are among the known adhesins that confer the adhesive properties to pathogenic Gram-positive bacteria. To investigate the role of CWAP of non-pathogen Gram-positive bacteria in the initial steps of biofilm formation, we evaluated the physico-chemical properties and adhesion to solid surfaces of *Lactococcus lactis*. To be able to grow in milk this dairy bacterium expresses a cell wall anchored proteinase PrtP for breakdown of milk caseins.

**Results:**

The influence of the anchored cell wall proteinase PrtP on microbial surface physico-chemical properties, and consequently on adhesion, was evaluated using lactococci carrying different alleles of *prtP*. The presence of cell wall anchored proteinase on the surface of lactococcal cells resulted in an increased affinity to solvents with different physico-chemical properties (apolar and Lewis acid-base solvents). These properties were observed regardless of whether the PrtP variant was biologically active or not, and were not observed in strains without PrtP. Anchored PrtP displayed a significant increase in cell adhesion to solid glass and tetrafluoroethylene surfaces.

**Conclusion:**

Obtained results indicate that exposure of an anchored cell wall proteinase PrtP, and not its proteolytic activity, is responsible for greater cell hydrophobicity and adhesion. The increased bacterial affinity to polar and apolar solvents indicated that exposure of PrtP on lactococcal cell surface could enhance the capacity to exchange attractive van der Waals interactions, and consequently increase their adhesion to different types of solid surfaces and solvents.

## Background

In natural aquatic populations, bacteria often live in biofilms, which may be described as matrix-enclosed bacterial communities attached to a substratum [1,2]. Biofilm formation allows bacteria to survive in environments that would be lethal for their planktonic counterparts [3,4]. Key event in biofilm formation is bacterial adhesion on a surface that depends on factors such as preconditioning of the support by macromolecules and the physico-chemical interactions between the bacterial cells and the substratum [5,6].

In the dairy industry, biofilms usually occur on surfaces that are in contact with fluids, and may be a source of bacterial contamination leading to technological and economical problems [7-9]. Nevertheless, protective biofilm formation on food industry workshop surfaces can also be beneficial because their presence may effectively modify the physico-chemical properties of substrates and as such, reduce adhesion of the undesirable planktonic microorganisms [10,11]. Furthermore, multiplication of the undesirable organism may be inhibited by nutrient competition or by synthesis of antagonistic compounds such as acids, bacteriocins, or surfactants [12,13]. In recent years, biofilms of lactic acid bacteria have received considerable attention for their potential use in the settlement of a competitive flora [14,15]. *Lactococcus lactis *is the most frequently used dairy bacterium for fermentation and preservation purposes. Lactococci do not present any detrimental effect on the sensory properties of processed foods, making them a suitable candidate for the creation of protective biofilms.

Various studies have demonstrated that bioadhesion depends mainly on combination of surface physico-chemical properties (such as Lewis acid-base character, capacity to exchange attractive van der Waals interactions, and global surface charge) of both the cell and the solid substratum [5,16,17]. Concerning bacterial surfaces, these properties depend on molecular cell surface composition. It was shown that the *L. lactis *ssp. *lactis *LMG9452 surface is composed mainly of proteins and polysaccharides and has a hydrophilic character [18]. However, it is still unclear as to which lactococcal cell surface molecules influence particular physico-chemical properties and adhesion.

Cell wall anchored proteins (CWAP) are among the known bacterial cell surface components having adhesive properties[19]. This group includes adhesins or proteins influencing coaggregation, e.g., fibronectin and collagen binding proteins of *Staphylococcus aureus*, *S. schleiferi *[20,21], or glucan binding protein of *Streptococcus mutans *[19]. Concerning *L. lactis*, three surface proteins were attributed to the same group of CWAP: *i) *the chromosomally-encoded sex factor CluA [22], *ii) *the plasmid-encoded proteinase NisP [23], and *iii) *the plasmid-encoded cell serine proteinase PrtP (also called lactocepin [24], which initiates proteolytic degradation of milk casein [25]). Like other CWAP, the lactococcal PrtP proteinases are characterized by a signal sequence at the N-terminus that is cleaved during secretion across the membrane; and a LPXTG sorting motif followed by a hydrophobic membrane-spanning region and a positively charged tail at the C-terminus [25]. After protein translocation through the membrane, the sortase enzyme mediates cleavage of LPXTG such that the threonine carboxyl group is linked to the cross-bridges in the peptidoglycan layer [26]. Deletion of the N-terminal end containing the LPXTG motif results in complete secretion of the truncated proteinase [27]. Fusion of the C-terminal LPXTG containing domain of PrtP with several reporter proteins resulted in the surface exposure of the fusion proteins [28,29].

The role of bacterial cell wall anchored proteins in adhesion was studied mainly in connection with their possible roles in virulence [21]. Previous studies addressed specific binding to host cell components like platelets, albumin, fibrinonectin, or collagen [20,21,30]. However, the role of cell wall anchored proteins of non-pathogenic bacteria on cell surface physico-chemical properties and adhesion to inert surfaces has not been examined.

The aim of this work was to evaluate the influence of the proteinase PrtP on hydrophobic/hydrophilic characteristics, Lewis acid-base properties, electrical charge and adhesive capacity of lactococci.

## Results

### Determination of the hydrophobic/hydrophilic and Lewis-acid base characters

We used derivatives of *L. lactis *ssp. *cremoris *strain MG1363: PRTP^+ ^(PrtP anchored and active), PRTP* (PrtP anchored and inactive) and PRTP^- ^(MG1363 carrying vector plasmid pGKV2 without *prtP *gene) as control strain. The strain MG1363 does not express other surface exposed proteinases although several membrane and cytoplasmic proteases are present [31]. As previously was shown that expression of various proteinase derivatives from the same promoter resulted in the same amount of proteinase [32], it was assumed that the proteinase expression in PRTP^+ ^and PRTP* strains was identical.

**Figure 1:**
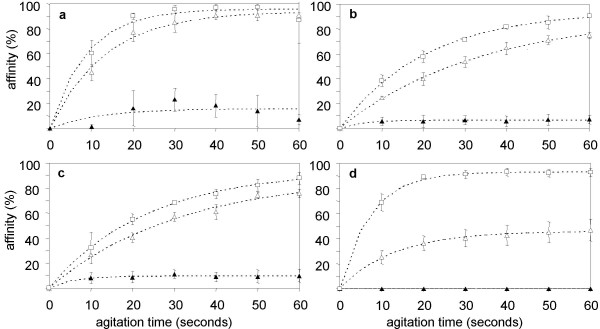
**Affinities of MG1363 derivatives carrying different *prtP *alleles to four solvents used in kinetic MATS analysis: chloroform (a), hexadecane (b), decane (c) and ethyl acetate (d)**. Open squares – PRTP*, open triangles – PRTP^+^, closed triangles – PRTP^-^.

The MATS kinetic experiment was used to determine the dynamic interaction of lactococci carrying different alleles of *prtP *gene (PRTP^-^, PRTP^+ ^and PRTP*) with polar (chloroform and ethyl acetate) and apolar (hexadecane and decane) solvents (Fig. 1). To extract the maximal affinity to solvents (*A*_*max*_) and the initial slope (*A*_*max*_·*k*) values, the experimental data presented in Fig. 1 were fitted using the following exponential expression: *A*(*t*) = *A*_*max *_·[1 - *e*^(-*k*·*t*)^]

where *A*(*t*) is the affinity as a function of time, *A*_*max*_, the maximal affinity; *A*_*max*_·*k*, the initial slope and *t*, the time in seconds. The maximal affinity and the initial slope values are presented in Table 1.

For cells expressing anchored proteinase PRTP* and PRTP^+ ^a maximum affinity was reached between 20 to 40 second interaction with mono-polar solvents (chloroform and ethyl acetate), while the maximum affinity to apolar solvents (hexadecane, decane) was attained after a period of time superior to 60 seconds. PRTP* had higher initial slope (12. 1) of affinity to ethyl acetate compared to PRTP^+ ^(3. 2). The difference between these two strains was slightly less pronounced in case of chloroform: 10.5 for PRTP* and 6.6 for PRTP^+ ^(Table 1).

Our results showed that control strain PRTP^- ^exhibited very low affinity for all four solvents (maximal affinity <20%) independently of their different physico-chemical properties (whether apolar, Lewis-acid or Lewis-base). Low affinity for apolar solvents (i.e. *A*_*max *_for hexadecane and decane was less than 10%), indicated the lack of hydrophobic properties of PRTP^- ^control strain (Table 1).

The hydrophobic character of the two other strains expressing anchored proteinase (PRTP^+ ^and PRTP*) was different: they both exhibited higher affinity to all solvents (P < 0.05; Fig. 1). The higher affinity for all solvents was observed in strain PRTP*, encoding anchored inactive PrtP. The presence of anchored proteinase generally resulted in an increase of bacterial affinity for apolar solvents hexadecane and decane, since *A*_*max *_values comprised in the range 89–95% for PRTP^+ ^and PRTP*, in comparison to values of less than 10% for control strain PRTP^- ^(P < 0.05; Table 1). This suggests that anchored PrtP, active or not, markedly mediated the increase of cell hydrophobicity.

### Evaluation of cell wall electrical charge

**Figure 2:**
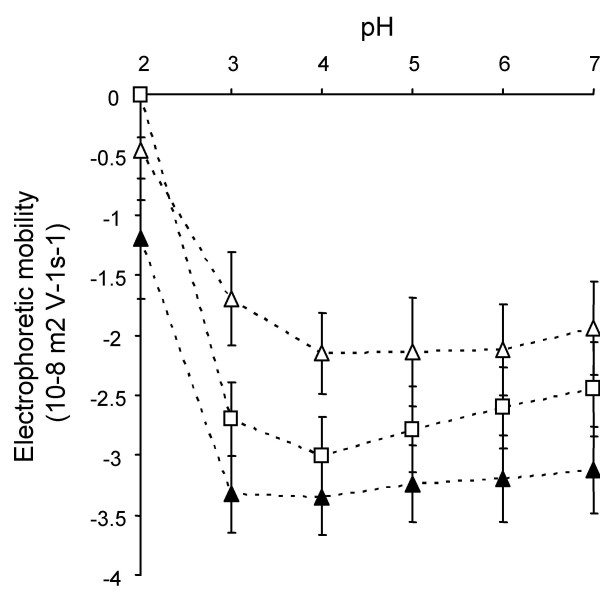
**Electrophoretic mobility of MG1363 derivatives carrying different *prtP *alleles in 1.5 mM NaCl solution at pH 2 to 7**. Open squares – PRTP*, Open triangles – PRTP^+^, closed triangles – PRTP^-^.

The same *L. lactis *strains, carrying different *prtP *alleles were used to evaluate global cell surface charge. Electrophoretic mobility (EM) of three bacterial strains (PRTP^-^, PRTP^+^, and PRTP*) at pH values ranging from 2 to 7 are presented in Fig. 2. We observed that all strains were highly electronegative and an isoelectric point could not be determined in the pH range explored. In all cases the EM values reached their minimum at pH values of 4, as was observed in previous studies with different lactic acid producing bacteria [18,33]. At pH range exceeding 3 the presence of anchored proteinase significantly reduced the negative charge of microbial cells (P < 0.05, Fig. 2). This effect was maximal for cells expressing anchored active proteinase: EM values of PRTP^+ ^were higher than -2 × 10^-8 ^m^2^V^-1^s^-1^, in comparison to less that -3 × 10^-8 ^m^2^V^-1^s^-1 ^for control strain PRTP^- ^(Fig. 2).

### Evaluation of adhesion to solid surfaces

We used glass and PTFE to study the influence of anchored PrtP on lactococcal adhesion to solid surfaces. Physico-chemical properties of these two solid substrates were evaluated by contact angle measurements. The van der Waals (γ^LW^), Lewis-base (γ^-^) and Lewis-acceptor (γ^+^) components of the surface tension (γ^S^) of glass and PTFE are presented in Table 2. In agreement with previously published data [6], glass exhibited a strong hydrophilic character (Θ_water _= 10°). The hydrophilic glass nature is mainly due to its Lewis base character (γ^- ^= 55 mJ·m^2^). This test indicated that PTFE was almost apolar (γ^AB ^~ 0) and exhibited very low van der Waals character (γ^LW ^= 15), indicating low interacting capacity.

Adhesion to glass and PTFE of lactococci expressing different *prtP *alleles was examined in two concentration NaCl, 1.5 mM and 150 mM. We observed a statistically significant increase (P < 0.05) of adhesion for strains expressing anchored PrtP, independently of their proteolytic activity and the surface (3 – 6 fold for PRTP^+ ^and 8 – 10 fold for PRTP*; Table 3). This increase was significantly (P < 0.05) higher in 150 mM NaCl solution.

## Discussion

The aim of this work was to study the involvement of the cell wall proteinase PrtP on physico-chemical mechanisms of adhesion of *L. lactis *to solid surfaces. In our experimental conditions, the presence of CWAP PrtP, active or inactivated, on the cell surface modified the physico-chemical surface properties as well as microbial adhesion to hydrophobic (PTFE) or hydrophilic (glass) surfaces (proteinase is active in PRTP^+ ^and inactive in PRTP*). Efficient adhesion of the strain expressing inactivated cell surface-anchored PrtP indicated that the presence of PrtP on the cell surface, and not its proteolytic activity, is important in this phenomena.

We ruled out possible effects of vector itself on adhesion: the physico-chemical properties and adhesion of MG1363 with or without vector pGKV2 [34], used to clone *prtP*, were essentially the same (results not shown). Proteolytic activity of cloned PrtP proteinases used in this work is comparable to that of a wild type strain, suggesting that their expression and anchoring could be also comparable [35]. This allows us to suggest that adhesion *via *PrtP may also occur in natural strains. Moreover, it has been shown that PrtP expression in milk is more efficient than in M17 medium, used in this study [31]. Therefore we can expect that in dairy environment the effect of PrtP on cell surface properties would be even more pronounced.

The adhesive behavior of strains bearing surface-anchored PrtP could be explained by changes in cell surface physico-chemical properties. Electrophoretic mobility measurements revealed that the presence of proteinase on the lactococcal cell surface is correlated with a reduced global negative charge. The high negative charge and the absence of isoelectric point in the pH range we examined could be linked to the presence of (lipo)teichoic acid in the cell wall that contains many phosphates groups with a pKa of around 2 [18]. The clear reduction of negative charge in cells displaying PrtP may be explained by an increase of the N/P (protein/phosphate) ratio of the bacterial cell wall [18]. The ability of PrtP to bind cations such as Ca^++ ^may also have an influence on global surface charge [36].

We observed more efficient adhesion of PRTP* strain to solid (glass and PTFE) surfaces as compared to PRTP^+ ^strain (p < 0.05; Table 3). Moreover, we observed the difference in PRTP*adhesion between high (150 mM) and low (1.5 mM) ionic strength conditions. Since both bacterial (Fig. 2) and glass or PTFE [37] surfaces are negatively charged, this could be explained by stronger electrostatic repulsion in low salt concentration. However, the differences in adhesion between PRTP^- ^and PRTP* strains were more expressed in high salt concentration, the conditions where repulsive electrostatic interactions are strongly diminished ([6], Table 3). We therefore suggest that electrostatic interactions do not play a predominant role in PrtP mediated adhesion.

The MATS test showed that strains bearing anchored PrtP had increased affinity for all solvents tested, independently of their nature, i.e., polar, less hydrophobic (ethyl acetate and chloroform) or apolar, more hydrophobic (decane and hexadecane). Furthermore, adhesion of strain bearing anchored PrtP increased regardless of whether the substrate was PTFE, which is apolar and hydrophobic, or glass, which is polar and hydrophilic [38]. Based on these results, we hypothesize that the presence of PrtP increases the capacity of the cell to exchange attractive van der Waals interactions; these interactions would increase bioadhesion of lactococci displaying anchored PrtP to different types of surfaces (e.g., inert, polar or apolar, or organic).

The affinity of inactive PRTP* to solvents and to solid surfaces was higher in comparison with its active counterpart PRTP^+^. This effect could be explained by degradation of main lactococcal autolysin AcmA by PRTP^+ ^[39]. AcmA activity was reported to increase significantly bacterial adhesion [40,41]. Degradation of AcmA by PrtP could diminish its activity and consequently adhesive properties. Alternatively, the greater affinity of inactive PrtP carrying strains to solvents and to solid surfaces may be explained by the absence of self-cleavage. Such self-cleavage is characteristic to an active proteinase and consequently could result in lower number of molecules present on cell surface [34].

We observed a very low affinity of lactococci to apolar solvents, consistent with previous results using *L. lactis *strain LMG9452 [18]. The presence of anchored proteinase thus increased strain hydrophobicity. The hydrophobic character was reported as feature of a number of Gram-positive bacteria which possess cell wall anchored proteins [42,43]. The increase of hydrophobicity by cell wall anchored proteins may be a common property of Gram-positive bacteria. Nevertheless, other factors (like polysaccharides) could mask this effect. For example, in the case of hydrophilic *L. lactis *strain LMG9452, the surface is dominated by polysaccharides rather than proteins [18].

Surface proteins other than those that are anchored via an LPXTG motif may affect bioadhesion. For example, autolysins of *Staphylococcus epidermidis *were recently shown to affect primary attachment to solid surfaces, and the autolysin of *Listeria monocytogenes *contributes to adhesion to eucaryotic cells [44,45]. Presence of PrtP on the lactococcal cell surface increases adhesion to glass and to PTFE about 10 fold. The ability of a single protein to change adhesion to this extent may also indicate that there are few other proteins present on the lactococcal cell surface or that these proteins do not affect adhesion. Two confirmed lactococcal proteins with cell wall anchor domains are the sex factor protein CluA [22], and plasmid-encoded NisP [23], which is not present in MG1363. The CluA dependent cell aggregation phenotype is reportedly poorly expressed unless a co-integrate is formed between the sex factor and a lactose plasmid [22], so we consider it unlikely that CluA is a significant adhesion factor in our experimental system.

## Conclusion

We have shown that the cell wall anchored PrtP proteinase, in addition to its role in milk casein degradation, is responsible for greater cell hydrophobicity and adhesion to solid surfaces. An increase of adhesion to polar and apolar solid surfaces and solvents indicates that attractive van der Walls interactions may be responsible for PrtP mediated lactococcal adhesion. Obtained results indicate that PrtP, and not its proteolytic activity, are responsible for the changes of these cell surface physico-chemical properties. We suggest that PrtP or its derivatives can be used as a tool to construct strains with increased adhesion that form protective biofilms.

## Methods

### Bacterial strains and growth conditions

The *Lactococcus lactis *ssp. *cremoris *strain MG1363 [46] was used as host for three isogenic plasmids: pGKV2 [47]; strain carrying this plasmid called here PRTP^-^); pGKV552 (derivative of pGKV2, containing cloned *prtPI *gene [34]; strain carrying this plasmid is called here PRTP^+^); and pGKV1552 (derivative of pGKV552, where PrtPI is inactivated by in-frame point mutation of Asp-30 to Asn-30 in a catalytic site [34], strain carrying this plasmid called here PRTP*). Plasmid pGKV2 contains the replication origin of the cryptic *L. lactis *WG2 plasmid pWV01 and the erythromycin and chloramphenicol resistance genes [47]. Bacteria were cultivated in M17 medium [48] supplemented with 5% of glucose at 30°C. When needed, 5 μg/ml of erythromycin was added.

### MATS (Microbial adhesion to solvents)

The method is based on comparing the affinity between microbial cells and a mono-polar or an apolar solvents [49]. The polar solvent can be an electron-acceptor or an electron-donor. The solvents used in this study were: chloroform (an electron-acceptor solvent), hexadecane (nonpolar solvent), ethyl acetate (an electron-donor solvent) and decane (nonpolar solvent). To evaluate kinetic of bacterial adhesion to solvents over night grown bacteria were harvested by centrifugation (7000 *g*, 4°C, 10 min.), then washed twice using 150 mM NaCl and a re-suspended in a 150 mM NaCl solution. The high NaCl concentration was used to avoid charge interference. The initial optical density (OD_i_) of this suspension was then adjusted to around 0.8 at 400 nm. The suspension was divided in six 2.4 ml samples, 0.4 ml of a solvent was added to each of them. The samples were mixed 10, 20, 30, 40, 50 and 60 seconds with agitator type vortex (Heidolph, Schwabach, Germany). The mixtures were allowed to stand for 15 min. for complete phase separation. The aqueous phase was then removed and the final optical density (OD_f_) was measured. The microbial adhesion to each solvent was calculated as (OD_i _- OD_f_)/OD_i _× 100 and presented in percents. Each experiment was performed in triplicate using independently prepared cultures.

### Electrophoretic mobility

After overnight growth, bacteria were harvested by centrifugation (7000 *g*, 4°C, 10 min.), washed twice with 1.5 mM NaCl and suspended in the same buffer at a final cell density of 10^7 ^cfu/ml. The pH of the suspension was adjusted in the range of pH 2 to 7, as needed, by adding nitric acid or potassium hydroxide (Sigma, Saint-Quentin, France). Electrophoretic mobility was measured with an automated zetameter (Zetaphoremètre II, CAD Instrumentations, Paris, France) using an electric field of 50 V. Each experiment was performed in triplicate using three independent cultures.

### Preparation of solid supports

The solid surfaces used in this experiment were micro cover glasses (Menzel Glass^®^, LDS 2460, 24 × 60, Braunschweig, Germany) and 3 × 1.4 cm coupons of polytetrafluorethylene (PTFE, Goodfellow SARL, Lille, France). Before adhesion tests, the supports were washed 15 min. at 50°C with detergent RBS 35 (2%, Société des Traitements Chimiques de Surfaces, Lambersart, France) with shaking, then rinsed five times with 50°C water and five times with Milli-Q water (Millipore, Saint-Quentin-en-Yvelines France).

### Contact angle measurements

The Lifshitz-van der Waals (γ^LW^), electron-donor (γ^-^) and electron-acceptor (γ^+^) surface tension components of the solid surfaces (S) were determined by measuring contact angles using the expression cos⁡θ=−1+2(γsLWγLLW)1/2/γL+2(γs+γL−)1/2/γL+2(γs−γL+)1/2/γL
 MathType@MTEF@5@5@+=feaafiart1ev1aaatCvAUfKttLearuWrP9MDH5MBPbIqV92AaeXatLxBI9gBaebbnrfifHhDYfgasaacH8akY=wiFfYdH8Gipec8Eeeu0xXdbba9frFj0=OqFfea0dXdd9vqai=hGuQ8kuc9pgc9s8qqaq=dirpe0xb9q8qiLsFr0=vr0=vr0dc8meaabaqaciaacaGaaeqabaqabeGadaaakeaacyGGJbWycqGGVbWBcqGGZbWCiiGacqWF4oqCcqGH9aqpcqGHsislcqaIXaqmcqGHRaWkcqaIYaGmcqGGOaakcqWFZoWzdaqhaaWcbaGaem4CamhabaGaemitaWKaem4vaCfaaOGae83SdC2aa0baaSqaaiabdYeambqaaiabdYeamjabdEfaxbaakiabcMcaPmaaCaaaleqabaGaeGymaeJaei4la8IaeGOmaidaaOGaei4la8Iae83SdC2aaSbaaSqaaiabdYeambqabaGccqGHRaWkcqaIYaGmcqGGOaakcqWFZoWzdaqhaaWcbaGaem4CamhabaGaey4kaScaaOGae83SdC2aa0baaSqaaiabdYeambqaaiabgkHiTaaakiabcMcaPmaaCaaaleqabaGaeGymaeJaei4la8IaeGOmaidaaOGaei4la8Iae83SdC2aaSbaaSqaaiabdYeambqabaGccqGHRaWkcqaIYaGmcqGGOaakcqWFZoWzdaqhaaWcbaGaem4CamhabaGaeyOeI0caaOGae83SdC2aa0baaSqaaiabdYeambqaaiabgUcaRaaakiabcMcaPmaaCaaaleqabaGaeGymaeJaei4la8IaeGOmaidaaOGaei4la8Iae83SdC2aaSbaaSqaaiabdYeambqabaaaaa@6FA6@[6]. We measured the contact angles (θ) of glass and PTFE with three pure liquids (L), which were deionised water (Purit, Lormont, France), formamide and diiodomethane (Sigma, Saint-Quentin, France).

### Bacterial adhesion to solid surfaces

Slides were incubated in 30 ml of bacterial suspension (O.D_600 _= 0.8) in 1.5 and 150 mM NaCl solution in Petri plates for 1 hour, then rinsed five times (care was taken to prevent slides from drying between washes), and colored for 15 min. with 0.01% (w/v) acridine orange water solution (Sigma, St. Louis, MO). Fluorescently colored cells were visualized and images captured with epifluorescence microscope (Leica DMLB, Tokyo, Japan, equipped with objective 10×). Ten images of each slide were taken and analyzed with UTHSCSA ImageTool program . Microbial adhesion was estimated as the percentage of solid surface covered by bacteria. Each value presented is the mean of at least three independent set of experiments.

### Statistical analysis

Multifactor ANOVA variance analyses were performed with statistical analysis program Statgraphics Plus 4.1 (Manugistics, Rockville, MD).

## Authors' contributions

OH and CLG performed MATS, adhesion to solid surfaces and electrophoretic mobility measurements and helped in draft the manuscript, VJ and MNBF participated in the design of the study and interpretation of results, GB participated in plasmid constructions, design of the study and critical reading of the manuscript, SK and RB conceived the study and drafted the manuscript. All authors read and approved the final manuscript.

## Figures and Tables

**Table 1:** The maximal affinity to chloroform, hexadecane, decane and ethyl acetate and initial slope values of MG1363 carrying different *prtP *alleles

	**Strain**	**Chloroform**	**Hexadecane**	**Decane**	**Ethyl acetate**
A_*max*_	PRTP ^-^	15.6	6.5	9.6	0.0
	PRTP ^+^	94.0	90.2	89.5	45.9
	PRTP *	95.8	94.0	94.1	93.1

*A*_*max*_·*k*	PRTP ^-^	1.2	1.2	1.6	0.0
	PRTP ^+^	6.6	2.7	2.8	3.2
	PRTP *	10.5	3.8	4.0	12.1

The maximal affinity and initial slope values were extracted from experimental data presented in Fig. 1.

**Table 2:** Surface tension components of glass and PTFE

	**θ**_**W**_	**θ**_**F**_	**θ**_**D**_	**γ**^**LW**^	**γ**^**-**^	**γ**^**+**^	**γ**^**AB**^	**γ**^**S**^
Glass	10°	17°	61°	28	55	4	30	58
PTFE	109°	95°	83°	15	1.5	0	0	15

Contact angles measured on glass and PTFE with water (θ_w)_, formamide (θ_F) _and diiodomethane (θ_D_); and derived Van der Waals, (γ^LW^), electron-donor (**γ**^**-**^), electron-acceptor (**γ**^**+**^) and polar (**γ**^**AB**^) components of the materials surface energy (**γ**^**S**^) expressed in mJ·m^-2^.

**Table 3:** Adhesion to glass and PTFE surface by MG1363 carrying different *prtP *alleles in two NaCl concentrations.

Strain	Percentage of surface coverage on:			
	
	Glass		PTFE	
	
	150 mM NaCl	1.5 mM NaCl	150 mM NaCl	1.5 mM NaCl
PRTP^-^	5.0 ± 1.9	4.5 ± 1.5	4.7 ± 2.9	3.5 ± 1.7
PRTP ^+^	33.5 ± 8.4	18.4 ± 4.2	12.7 ± 3.4	13.6 ± 3.5
PRTP *	49.7 ± 6.8	35.8 ± 6.9	39.0 ± 7.5	22.7 ± 4.1
